# VIS832, a novel CD138-targeting monoclonal antibody, potently induces killing of human multiple myeloma and further synergizes with IMiDs or bortezomib in vitro and in vivo

**DOI:** 10.1038/s41408-020-00378-z

**Published:** 2020-11-02

**Authors:** Tengteng Yu, Bharat Chaganty, Liang Lin, Lijie Xing, Boopathy Ramakrishnan, Kenneth Wen, Phillip A. Hsieh, Andrew Wollacott, Karthik Viswanathan, Hedy Adari, Shih-Feng Cho, Yuyin Li, Hailin Chen, Wenjuan Yang, Yan Xu, Gang An, Lugui Qiu, Nikhil Munshi, Gregory Babcock, Zachary Shriver, James R. Myette, Kenneth C. Anderson, Yu-Tzu Tai

**Affiliations:** 1 Jerome Lipper Multiple Myeloma Center, LeBow Institute for Myeloma Therapeutics, Dana-Farber Cancer Institute, Harvard Medical School, Boston, MA USA; 2 State Key Laboratory of Experimental Hematology, National Clinical Research Center for Hematological Disorders, Institute of Hematology and Blood Diseases Hospital, Chinese Academy of Medical Sciences and Peking Union Medical College, Tianjin, 300020 China; 3 Visterra Inc., Waltham, MA USA; 4 Division of Hematology & Oncology, Department of Internal Medicine, Kaohsiung Medical University Hospital, Kaohsiung Medical University, Kaohsiung, 80708 Taiwan; 5 Faculty of Medicine, College of Medicine, Kaohsiung Medical University, Kaohsiung, 80708 Taiwan; 6 School of Biotechnology, Tianjin University of Science and Technology, Key Lab of Industrial Fermentation Microbiology of the Ministry of Education, State Key Laboratory of Food Nutrition and Safety, Tianjin, 300457 China; 7 Hematology Department, Yueyang Hospital of Integrated Traditional Chinese and Western Medicine, Shanghai University of Traditional Chinese Medicine, Shanghai, China

**Keywords:** Targeted therapies, Cancer

## Abstract

Therapeutically targeting CD138, a define multiple myeloma (MM) antigen, is not yet approved for patients. We here developed and determined the preclinical efficacy of VIS832, a novel therapeutic monoclonal antibody (MoAb) with differentiated CD138 target binding to BB4 that is anti-CD138 MoAb scaffold for indatuximab ravtansine (BT062). VIS832 demonstrated enhanced CD138-binding avidity and significantly improved potency to kill MM cell lines and autologous patient MM cells regardless of resistance to current standard-of-care therapies, via robust antibody-dependent cellular cytotoxicity and phagocytosis mediated by NK and macrophage effector cells, respectively. Specifically, CD38-targeting daratumumab-resistant MM cells were highly susceptible to VIS832 which, unlike daratumumab, spares NK cells. Superior maximal cytolysis of VIS832 vs. daratumumab corresponded to higher CD138 vs. CD38 levels in MM cells. Furthermore, VIS832 acted synergistically with lenalidomide or bortezomib to deplete MM cells. Importantly, VIS832 at a sub-optimal dose inhibited disseminated MM1S tumors in vivo as monotherapy (*P* < 0.0001), and rapidly eradicated myeloma burden in all mice concomitantly receiving bortezomib, with 100% host survival. Taken together, these data strongly support clinical development of VIS832, alone and in combination, for the therapeutic treatment of MM in relapsed and refractory patients while pointing to its potential therapeutic use earlier in disease intervention.

## Introduction

Multiple myeloma (MM) is characterized by excess monoclonal plasma cells (PCs) in the bone marrow (BM) producing monoclonal immunoglobulins, and is associated with hypercalcemia, renal dysfunction, anemia, and osteolytic bone disease^1,2^. Despite the recent use of novel therapies including proteasome inhibitors, i.e., bortezomib (btz), immunomodulatory drugs (IMiDs), i.e., lenalidomide (len) and pomalidomide (pom), and monoclonal antibodies (mAbs), i.e., daratumumab (dara) and isatuximab targeting CD38, alone or in combination, MM remains an incurable disease in most patients due to the development of drug resistance underlying relapse of disease^3,4^. Thus, it is urgent to develop well tolerated novel targeted immunotherapies to treat relapsed and refractory (RR) MM, which can then be used to treat earlier stages of disease and further improve patient outcome.

CD138 (Syndecan-1, SDC1), a member of the integral membrane family of heparan sulfate proteoglycans, is highly expressed on differentiated PCs. It is overexpressed in patient MM cells compared with normal PCs, and a validated diagnostic biomarker of MM. Importantly, CD138 is a co-receptor for MM cell growth, adhesion, and survival^5–8^, as well as other critical aspects of myeloma biology^9–13^. Its expression further correlates with disease progression and prognosis^14–16^. Thus, CD138 is a promising antigen for mAb-based immunotherapy of all stages of MM. Although CD138 targeted agents have and will be developed first to treat RRMM, CD138 is also expressed on smoldering myeloma (SMM), prior to the development of MM without evidence of end-organ damage^17–19^. Indeed, several clinical trials of MM therapies are now under evaluation to delay the progression of SMM to active disease^20–24^. Such therapies must not only delay progression of disease, but also be well tolerated, since these patients are asymptomatic.

To date, targeting CD138 for treatment of MM was clinically evaluated using indatuximab ravtansine (BT062), an anti-CD138 mAb (BB4) drug conjugate (ADC) specifically delivering a cytotoxic maytansine derivative to MM cells^25^. Indatuximab ravtansine showed encouraging anti-MM activity with a reasonable safety profile in a recent Phase Ib/IIa open label, multi-dose escalation, clinical trial in RRMM^26^. In a preclinical study, CD138-based chimeric antigen receptor T cells (CAR-T) also demonstrated significant killing of MM cells, without off tumor cytotoxicity against normal epithelial or endothelial cells^27^. An anti-CD138 mAb-based therapy with potent immune-mediated cytotoxicity could represent an effective and safer modality compared to an ADC or CAR-T. Both ADC and CAR-T approaches have potential liabilities or limitations, e.g., dose-limiting toxicities in the case of ADCs or a less durable clinical response, cytokine release syndrome, prohibitive cost of manufacture, and limited point of patient access in the case of CAR-T.

We here developed a novel anti-CD138 mAb VIS832 and characterized its mode of actions in multiple preclinical models of MM. In concert with its effective immune-mediated killing of MM cells as a single agent, in vitro and in vivo evaluations of VIS832 combined with len or btz demonstrated augmented efficiency and tolerability, providing the rationale for its clinical evaluation in MM.

## Materials and methods

### CD138 antibodies

VIS832 was produced by transient vector transfection in Chinese hamster ovary cells, as described in Supplementary Methods. The secreted mAb was purified from cell culture media using protein A affinity capture on Fast Protein Liquid chromatography (FPLC), in accordance with established protocols.

### Antibody-dependent cellular cytotoxicity (ADCC) assay

ADCC activity was mainly measured based on the calcein-AM release from calcein-AM labeled target MM cells co-incubated with human NK or PBMC effector cells isolated from multiple healthy donors or MM patients, in the presence of test Abs. Unless otherwise noted, target MM cells and NK effector cells were co-cultured at an effector: target (E:T) ratio of 10:1. ADCC data following antibody titration were plotted using a nonlinear regression analysis and a 4-parameter curve fit (GraphPad), from which EC_50_ values were calculated.

Bioluminescence (BLI)-based assays were also used when target MM cell transfectants expressing a luciferase gene were used^28^. When determining VIS832-induced ADCC against autologous patient MM cells in BMMCs from patient BM samples, flow cytometry-based analysis gated on viable BCMA+ fraction was used.

### Antibody-dependent cellular phagocytosis (ADCP) assay

Indicated target MM cell lines (*n* ≥ 5) sensitive or resistant to dexamethasone (dex), IMiDs, or dara were labeled with a fluorescence cell membrane dye Cell Trace™ Far Red, washed, and incubated for 4 h with macrophages labeled green with CFSE in the presence of VIS832 or antibody isotype control in triplicate at indicated E:T ratios (1:1, 2:1, or 4:1). Cells were fixed with 1% paraformaldehyde followed by flow cytometry to detect double-positive (DP) fluorescence, an indication of phagocytic activity^29^.

### Murine model of disseminated human MM disease

The in vivo efficacy of VIS832 and btz, alone or in combination, were evaluated using a murine model of the dissemination of MM1S-luc cells intravenously (i.v.) injected into CB-17 SCID mice. After 14 days, whole body tumor burden was monitored by BLI. Mice (*n* = 9 per group) were randomized and treatments started. Vehicle control (PBS) was dosed once daily by intraperitoneal (i.p.) injection (0.2 ml/20 g per mouse); VIS832 (4 mg/kg) and btz (1 mg/kg) were dosed twice weekly by i.p. injection for 52 days. Treatment in all groups was discontinued at Day 53; surviving animals were monitored for an additional 3 weeks till Day 73.

See Supplementary “Materials and Methods” for additional details.

## Results

### Robust binding of VIS832 to CD138 on MM cell lines and patient MM cells

CD138 protein and mRNA levels were first assayed by flow cytometry analysis using an anti-CD138 DL-101 clone and qRT-PCR, respectively, in MM cell lines (*n* = 12) including paired sensitive and resistant to dex (MM1S/MM1R), IMiDs (both len and pom) (MM1S/MM1S(R), H929/H929(R)), and btz (ANBL6/ANBL6-BR)^28,30^ (Supplementary Fig. S1A). All MM cell lines highly expressed CD138, as shown by geometric mean fluorescence intensity (MFI) values, with some variability in relative expression levels. MM cell lines (*n* = 29) expressed highest CD138 levels, compared with 10 other hematological cancer cell line cohorts (Supplementary Fig. S1B). Soluble CD138 generally correlated with protein and transcript levels in most of the corresponding MM cell lines (Supplementary Fig. S1C) and was elevated in patient serum samples vs. healthy donors (*P* < 0.001) (Supplementary Fig. S1D).

**Fig. 1:**
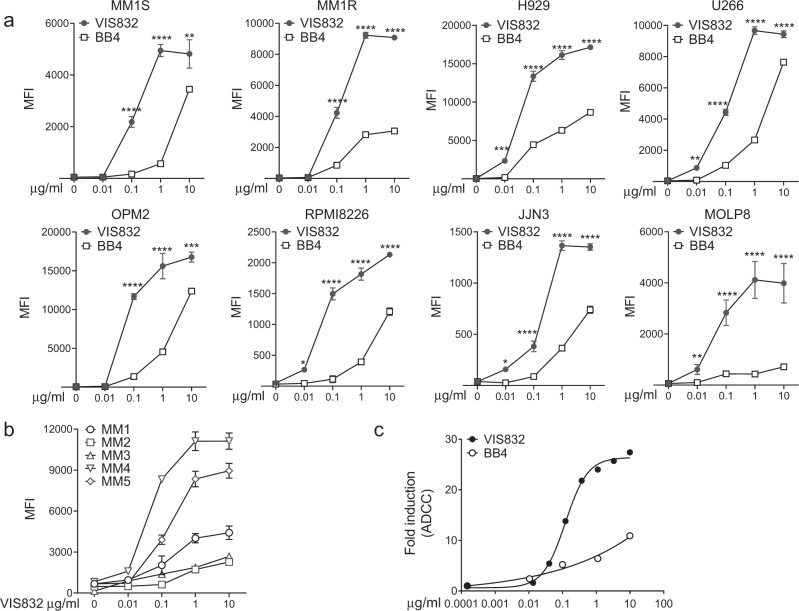
Binding of VIS832 was significantly more intense than BB4 to CD138 on MM cell membrane, potentiating FcR-mediated MM cell cytotoxicity. **a** Indicated MM cell lines (*n* = 8) were incubated with a serial dilution of VIS832 or BB4 (0–10 μg/ml) followed by flow cytometry (FC) analysis using an APC-conjugated secondary anti-human IgG1 antibody for detection. Binding intensity was reported as geometric mean fluorescence intensity (MFI) normalized to isotype control. Isotype control for VIS832 and BB4 (10 μg/ml) were used as background controls. Three experiments were done in triplicates for each concentration. Data were presented as means (MFIs) ± standard errors of means (SEMs, error bars). **P < 0.01, ***P < 0.005, ****P < 0.001. **b** Bone marrow mononuclear cells (BMMCs) from five patients (MM1-5) were incubated with VIS832 (0–10 µg/ml). Cells were subsequently co-stained with PE-BCMA antibody (to identify patient myeloma cells) and APC-anti-human IgG1 followed by FC analysis on PE + APC + cells. All data is presented as means (MFIs) ± SEMs (error bars) of triplicates at each VIS832 concentration. **c** Relative antibody-dependent cellular cytotoxicity (ADCC) of VIS832 vs. *BB4* possessing an isotype matched human IgG1 were measured using an ADCC reporter cell assay where Jurkat cells stably transfected with human FcRIIIa as surrogate for effector cells were co-cultured with target U266 MM cells. ADCC is reported as a fold induction over no antibody background control (effector + target cells only).

VIS832 is a humanized IgG1κ mAb targeting human CD138 and has been optimized for its binding avidity to cell membrane CD138. The epitope of VIS832 includes a membrane proximal region of CD138 predicted to confer productive immune synapse formation for improved Fc effector-dependent, immune cell-mediated cytotoxicity. The relative binding affinity of VIS832 to MM cells was compared with anti-CD138 antibody BB4, whose VH and VL variable regions comprise the paratope for indatuximab ravtansine^25,26^. VIS832 showed robust and dose-dependent target engagement, with potent binding avidity (sub-to low-nanomolar) across MM cell lines (*n* = 8), as indicated by MFI values (Fig. 1a). Significantly, binding of VIS832 was more intense (*P* < 0.005) than BB4 in all MM cell lines, with multi-fold increases in apparent binding affinity (Supplemental Table S1). Importantly, VIS832 showed dose- and CD138 target-dependent binding of PCs derived from BM of patients (4 RR and 1 SMM) (Fig. 1b). Thus, VIS832 is a potential therapeutic candidate targeting CD138 for further investigation in human MM.

### VIS832 directs target-specific ADCC against MM cell lines with acquired resistance to current therapies and in the presence of MM growth promoting BM cells

Antibody-dependent cellular cytotoxicity (ADCC) is an effective and therapeutically validated immune-based mechanism for targeted MM cell killing^29,31–36^. ADCC of VIS832 was first determined using a reporter bioassay comprised of an engineered Jurkat cell line overexpressing the human FcγRIIIa receptor as effector cell surrogate co-cultured with target U266 MM cell line. EC_50_ value for VIS832 was ~99.31 ng/ml (0.66 nM), whereas the *BB4* possessing an isotype matched human IgG1 induced only minimal ADCC (Fig. 1c). Afucosylation of the Fc N glycan on VIS832 further optimized its efficiency and potency, with decreased EC_50_ and >7-fold increased maximal U266 MM cell lysis (28.67 ng/ml (0.2 nM), Supplemental Fig. S2).

**Fig. 2:**
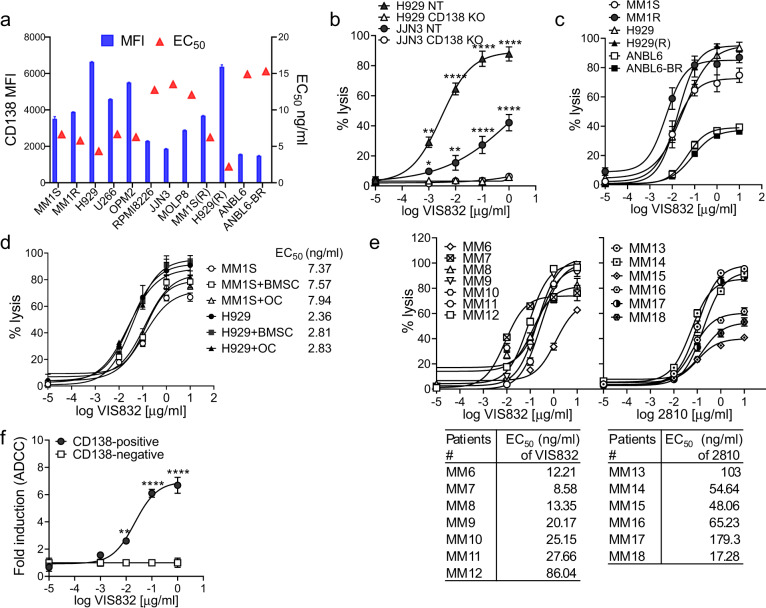
VIS832 specifically induced ADCC against MM cell lines and patient MM cells sensitive or resistant to current therapies in the presence or absence of BMSCs or OCs. **a** Human NK effector cells from 3 healthy donors were incubated with indicated MM cell lines (*n* = 12) (E:T = 10:1) in a serial concentration of VIS832 (0–10 μg/ml). VIS832-induced ADCC to lyse MM cells was determined using the calcein-AM release assay. Derived EC_50_ values (triangle, right label) were plotted in parallel with CD138 MFI (bar, left label). **b** VIS832-induced ADCC was done using target cells including parental (target control, NT) and CD138-knockout (KO) H929 and JJN3 cell lines. Parental H929 cells express higher CD138 than JJN3 cells. **c** Additional NK effector cells (*n* = 3) were co-incubated with target cells including paired MM1S and dex-resistant MM1R, H929, and len/pom-resistant H929(R), as well as ANBL6 and btz-resistant ANBL6-BR cells. **d** VIS832-induced ADCC against target MM1S or H929 MM cell lines was performed in the presence or absence of BMSCs or OCs. All data are presented as means (% lysis) ± SEMs (error bars) of three independent experiments using NK cells from three healthy donors with each performed in triplicate at each concentration. **e** BMMCs freshly derived from BM aspirates from patients (MM6-12, left; MM13-18, right) were treated with VIS832 (left) or mAb 2810 (right, a mouse-human chimeric antibody of VIS832) (0–10 μg/ml) for 24 h, followed by FC analysis to determine % lysis of patient MM cells. The % lysis indicated % depletion of viable BCMA+ patient PCs in BMMCs, and data was normalized to minus isotype control human IgG1 (set as 100% BCMA+ cells). BMMCs included target patient PCs, immune effector cells, and other non-MM BM accessory cells. **f** Calcein-AM-based ADCC assays were done using target cells including paired CD138-positive and CD138-negative cells separated from BMMC samples from three RRMM patients. Each target cell fraction was co-cultured with PBMCs from the same individual in the presence of VIS832 (0–1 µg/ml). Each experiment was performed in triplicate at each concentration and results are shown as means (% lysis) ± SEMs (error bars). *P < 0.05, **P < 0.01, ****P < 0.001.

Using calcein-AM release assay, VIS832 ADCC activity was next quantified in the co-cultures of calcein-AM-pre-labeled MM cell lines with NK cells from multiple healthy donors (*n* ≥ 3). VIS832, dose-dependently, induced ADCC against MM cell lines (*n* = 12) (Supplementary Fig. S3A). EC_50_ values of VIS832 ranged from 2.22 ± 0.37 to 15.3 ± 2.71 ng/ml, and % maximal lysis ranged from 37.06 ± 1.45% to 97.3 ± 3.34% across tested MM cell lines (Fig. 2a and Supplementary Table S2). VIS832-induced ADCC against MM cells generally correlated with CD138 surface expression levels (Supplementary Fig. S3B). VIS832-induced lower ADCC activity against target JJN3 (Fig. 2a, b) and ANBL6 (Fig. 2a, c) MM cells, presumably due to relatively lower CD138 expression (e.g., relative to levels in H929 or MM1S). VIS832-induced ADCC against MM cell lines was also consistent with ADCC activity induced by the parental mAb 2810, the non-humanized predecessor to VIS832 (Supplementary Fig. S3C). These comparable data (Supplementary Table S2, EC_50_ values ranging from 2.39 to 28.16 ng/ml across 10 MM cell lines) confirmed the successful antibody humanization of VIS832 with full retention of biological activity.

The dependency of CD138 expression on VIS832-induced NK cell-mediated MM cell lysis was validated using two CRISPR/CAS9-generated CD138 (SDC1) gene knockout (KO) H929 and JJN3 MM cell lines (Supplementary Fig. S3D). VIS832 did not induce ADCC against CD138 KO transfectants vs. the parental (target control, NT) cells with high (in H929) and low (in JJN3) CD138 levels (Fig. 2b).

VIS832 induced equivalent ADCC against paired MM cell lines sensitive and resistant to dex (MM1S/MM1R), len/pom-(H929/H929(R)), and btz (ANBL6/ANBL6-BR) (Fig. 2a, c). In the presence of BM stromal cells (BMSCs) or osteoclasts (OCs)^37–40^, two key MM-supporting accessory cells in the BM milieu, VIS832-induced ADCC against MM cells was minimally affected (Fig. 2d). These data indicated that VIS832 could generate potent cytotoxicity in MM cells resistant to current therapies and in the tumorigenic (and generally immunosuppressive) BM microenvironment.

### VIS832 effectively targets autologous patient MM cells

VIS832-dependent ADCC against autologous patient CD138 + cells was next evaluated using BMMCs freshly isolated from patient BM aspirates (*n* = 7, including 6 RRMM and 1 SMM (MM12)). BMMCs that included CD138 + MM cells, immune cells, and other BM accessory cells, were directly incubated with various dilutions of VIS832 for 1 day. The fraction of viable patient MM cells was independently measured by quantitative flow cytometry analysis after staining with an mAb against BCMA, another PC marker^41,42^. VIS832-induced autologous cytolysis of patient MM cells was determined by the elimination of the viable BCMA+ cell population relative to control (Supplementary Fig. S4). VIS832 depleted patient MM cells in a dose-dependent manner (Fig. 2e, left), inducing >60% maximal lysis of autologous patient MM cells, with >90% killing in 4 out of 7 MM patient samples. These results also replicated ADCC data in another RRMM patient cohort (*n* = 6, MM13-18) treated with the parental 2810 mAb, including 90% killing in 3 of 6 MM patient samples (Fig. 2e, right). EC_50_ values ranged from 8.58 to 86.04 and 17.28–179.3 ng/ml for VIS832 and 2810 mAb, respectively.

Paired CD138-positive and CD138-negative patient cell populations were next separated and pre-labeled with calcein-AM, followed by co-culture with autologous PBMCs (*n* = 3) in the presence of VIS832 (0–1 µg/ml). Specific ADCC activity was both VIS832-dependent and required CD138 + target patient MM cells (Fig. 2f). The correlation of MM cell binding and ADCC using VIS832 and 2810 mAb, therefore, corroborated effective CD138 target engagement, leading to productive and specific autologous patient MM cell killing.

### VIS832 showed increased ADCC potency than dara and directs significant target-specific killing of dara-resistant MM cells without depleting NK cells

**Fig. 3:**
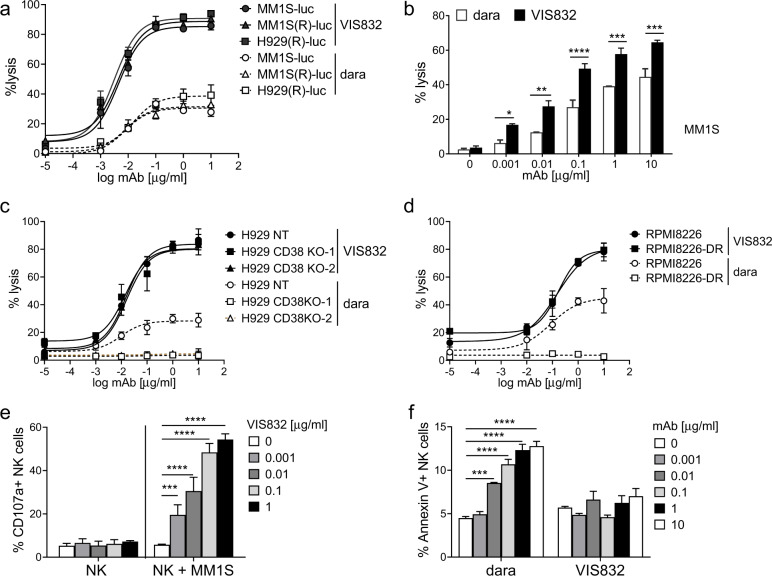
VIS832, unlike daratumumab, induced comparative ADCC against MM cells resistant to Dara- and IMiDs, without adverse effects on NK effector cells. **a**, **b** VIS832 showed a higher ADCC potency than dara, as measured by two orthogonal assays including BLI measurement (**a**) and quantitative FC analysis (**b**). **a** Target MM1S, MM1S(R), and H929(R) cells expressing firefly-luciferase (luc) were co-cultured with NK cells from three healthy donors in the presence of VIS832 or daratumumab (dara) (0–10 µg/ml). Luciferase activity in the cell culture media was quantified using the Dual-Luciferase® Reporter Assay Kit (Promega % cell lysis was normalized to 100% lysis of target cells set following mild detergent solubilization using assay compatible lysis buffer). **b** MM1S targeted cells were treated with VIS832 or dara in the presence of NK cells (*n* = 3 healthy donors) for 24 h followed by quantitative FC analysis to determine the % lysis (% of depleting viable BCMA+ cells). **c**, **d** VIS832- vs. dara-induced ADCC were determined in the 4-h calcein-AM release assay in co-cultures of indicated paired MM cell lines and NK cells from three healthy donors. Target MM cells included parental and two CD38 CRISPR-cas9 knockout (CD38KO-1, CD38KO-2) H929 (**c**) or dara-resistant RPMI8226 (PMIR8226-DR) (**d**) MM cells. Dara-resistant RPMI8226 cells were generated through extended exposure to a lower dose of dara with NK cells in the long-term culture. **e** NK cell activation was measured by double marker FC analysis (CD56 + and CD107a + ). CD107a cell surface expression was used as a surrogate for quantifying cellular degranulation of NK cells. NK cell activation was both VIS832- and target MM cell-dependent. **f** Effects of targeting CD38 vs. CD138 on NK cell viability. Fresh NK cells purified from healthy donors (*n* = 3) were incubated with serially diluted dara or VIS832 (0–10 µg/ml) for 24 h. NK cell apoptosis was assessed by Annexin V/PI staining followed by FC analysis. All assays included three independent experiments using NK cells from three healthy donors. Data are presented as means (% lysis) ± SDs (standard deviations, error bars). *P < 0.05, **P < 0.01, ***P < 0.005, ****P < 0.001.

We next evaluated relative potencies of VIS832 vs. daratumumab (dara), to induce effector cell-dependent killing of target MM cell lines using luminescence-based (Fig. 3a) and flow cytometry analysis (Fig. 3b). Dara targeting CD38 has recently been approved to treat newly diagnosed MM^43^. VIS832, more potently than dara, increased maximal lysis by ~3-fold of all target MM cell lines, regardless of resistance to both IMiDs. Higher MM1S cell lysis induced by VIS832 vs. dara was also confirmed in BCMA-based flow cytometry analysis (*P* < 0.05). These results confirmed significantly higher CD138 vs. CD38 levels in all MM cell lines (*P* < 0.0001, Supplementary Fig. S5A). Importantly, patient MM cells also expressed significantly higher CD138 vs. CD38 (*P* < 0.0001), and CD138 levels were further increased with progression of disease (Supplementary Fig. S5A, B).

We next asked whether VIS832 remained active against MM cells resistant to dara. CD38 knock-out (CD38 KO) through CRISPR/CAS9 gene editing was done in H929 cells to derive 2 CD38 KO transfectants (Supplementary Fig. S5C). VIS832 induced equivalent ADCC to lyse CD38 KO transfectants and the parental control H929 (H929 NT) cells (Fig. 3c). CD138 levels were unchanged in CD38 KO and parental control H929 cells. The dose response curves for three cell lines overlapped, with similar % maximal lysis and EC_50_ values of VIS832. Compared to dara, VIS832 induced significantly higher ADCC, with increased maximal lysis (~2.7-fold higher) of the parental H929 target cells. In contrast, dara failed to induce ADCC against CD38 KO H929 transfectants. We also developed dara-resistant RPMI8226 (RPMI8226-DR) cells via extended exposure of dara in ex vivo NK-MM co-cultures. Unlike RPMI8226 cells, dara did not induce ADCC against RPMI8226-DR cells, confirming resistance to dara (Fig. 3d). VIS832 still induced lysis of RPMI8226-DR to a similar extent as its parental RPMI8226 cells. Again, VIS832, more effectively than dara, induced greater % maximal lysis of the parental RPMI8226 cells.

The effect of VIS832 on MM-specific NK cell activation was next assayed by quantitative flow cytometry analysis for % CD107a surface expression on CD56 + CD3- cells in the NK-MM co-culture. VIS832 induced CD107a degranulation in NK cells in a MM cell- and concentration-dependent manners (Fig. 3e).

NK cells express CD38 at the highest level among other normal hematological lineage cells. In fact, dara depletes patient NK cells within the first week after treatment, affecting its efficacy to target MM cells and thereby limiting durability of responses^36,44^. Using annexin V staining followed by flow cytometry analysis to measure apoptotic cell fraction, dara, but not VIS832, induced NK cell death in a concentration-dependent manner after 1-day treatment (Fig. 3f). Thus, unlike dara, VIS832 even selectively induced MM cell lysis via NK activation, without depleting NK cells.

### Enhancement of targeted cellular killing activity of VIS832 in combination with lenalidomide or bortezomib

**Fig. 4:**
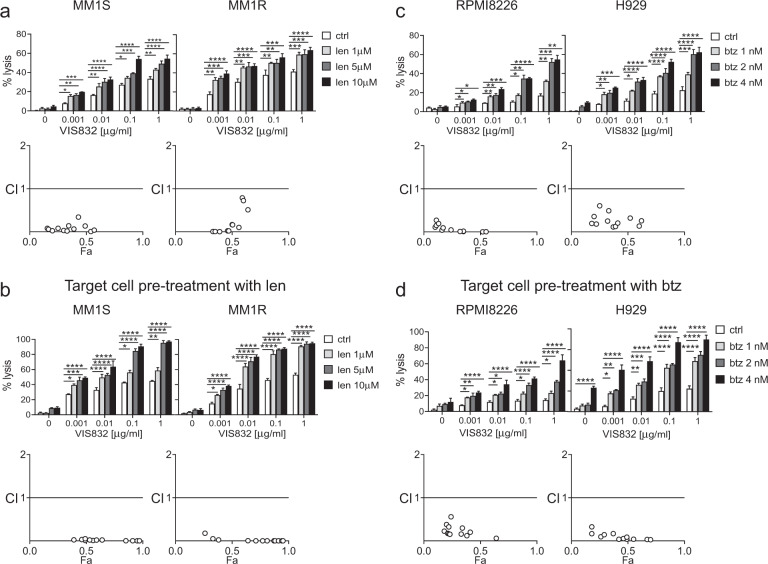
Lenalidomide and bortezomib enhanced VIS832-induced MM cell lysis in a synergistic manner. Indicated MM cell lines (**a**, **b** MM1S, MM1R; **c**, **d** RPMI8226, H929) were pretreated without (**a**, **c**) or with lenalidomide (len, **b**) or bortezomib (btz, **d**), and then drugs were washed out. Target MM cells were co-incubated with NK cells (E:T = 4:1) in the presence of varying concentrations of VIS832 or control media (ctrl) in the 2-h calcein-AM release assay. **a**, **c** VIS832 was added together with Len (**a**) or btz (**c**) in the co-cultures of target MM cells with NK cells. Percentages of MM cell lysis were used to calculate the combination index (CI) (Fa-CI plot) shown below each graph. CI < 1 indicated a synergistic interaction. Fa, fraction affected. All assays included three independent experiments using NK cells from 3 healthy donors. Data are presented as means (% lysis) ± SDs (error bars). *P < 0.05, **P < 0.01, ***P < 0.005, ****P < 0.001.

IMiDs (len and pom), which upregulate ADCC ability of therapeutic mAbs^29,32,34^, are routinely used in combination with dex in mAb-based immunotherapies in MM. Using calcein-AM release assays in MM-NK cell co-cultures at lower E:T ratio of 4:1, we next tested effects of len on VIS832-induced cytotoxicity against MM cells. When VIS832 and len were added concomitantly, synergistic MM cell lysis was seen, with combination index (CI) values of <1 at all concentrations of both drugs (Fig. 4a and Supplementary Fig. S6A). Pretreatment with len alone for 3 days did not induce significant killing of MM1S and MM1R target cell lines (<10%) (Fig. 4b). Also, VIS832 significantly induced higher ADCC against MM1S and MM1R target cells pretreated with len when compared to control medium groups (*P* < 0.05), with CI values <1 (Supplementary Fig. S6B).

Furthermore, VIS832-induced ADCC against target MM cell lines H929 and RPMI8226 was also synergistically enhanced when VIS832 combined with btz, at even lower E:T ratio of 1:1, with CI values of <1 (Fig. 4c, d and Supplementary Fig. S6C, D).

### VIS832 induced antibody-dependent cellular phagocytosis (ADCP) against MM cells sensitive and resistant to dex, IMiDs, or dara

**Fig. 5:**
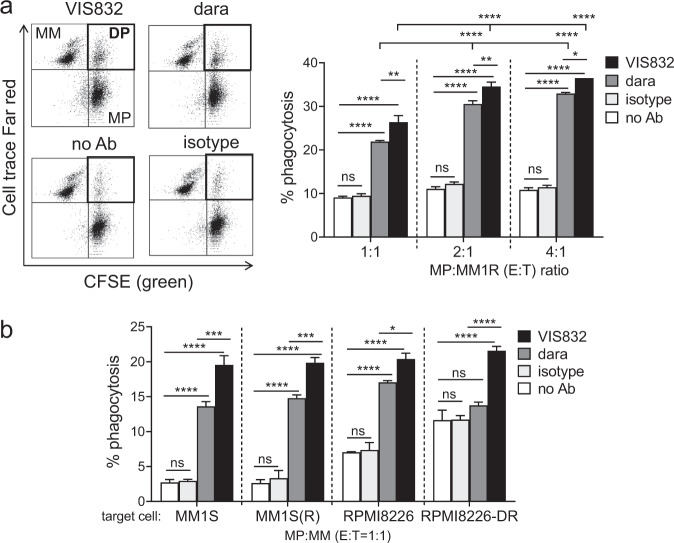
VIS832 induced antibody-dependent cellular phagocytosis (ADCP) against MM cells. **a** Target MM1R (MM) cells were pre-labeled with a fluorescence cell membrane dye Cell Trace™ Far Red and co-cultured for 4 h with macrophage (MP) effector cells pre-labeled with CFSE-FITC (green) in the presence of VIS832, daratumumab (dara), or isotype control (4.5 µg/ml each) at various E:T ratios of 1:1 to 4:1. Cells were fixed with 2% paraformaldehyde, followed by FC analysis to determine double-positive (DP) fluorescence fraction. Percent phagocytosis was determined as the number of DP cells (upper right panel) divided by the total number of target MM cells. Shown are representative dot plots at an E:T ratio of 2:1 (left) and summarized % phagocytosis at 3 E:T ratios (right). **b** Paired IMiDs- or dara-resistant target MM cell lines (*n* = 4) were co-incubated with MP cells (E:T = 1:1). All experiments were done in triplicates at each condition using MP cells derived from three healthy donors. ns, not significant; *P < 0.05, **P < 0.01, ****P < 0.001.

ADCP activity was next measured by dual colored flow cytometry analysis using in vitro culture-differentiated macrophages co-incubated with target MM1R cells resistant to dex. Percentage of phagocytosis as the double-positive (DP) fraction was quantitated in the presence of VIS832, isotype control Ab, or no Ab control, at various macrophage effector to MM cell ratios. Dara served as a positive control for its reported ADCP activity^45^. Phagocytosis was dependent on both VIS832 (e.g., in comparison to the isotype control antibody) and the E:T ratio (*P* < 0.001, Fig. 5a**)**. The ADCP activity of VIS832 was significantly higher than that observed for dara (*P* < 0.05, Fig. 5a, b). Furthermore, VIS832-induced ADCP was comparable in MM cell lines sensitive or resistant to IMiDs and dara (Fig. 5b). While dara failed to induce ADCP against RPMI822-DR cells, VIS832 still induced significant ADCP (*P* < 0.0001).

### VIS832 efficacy in a murine model of disseminated human MM

**Fig. 6:**
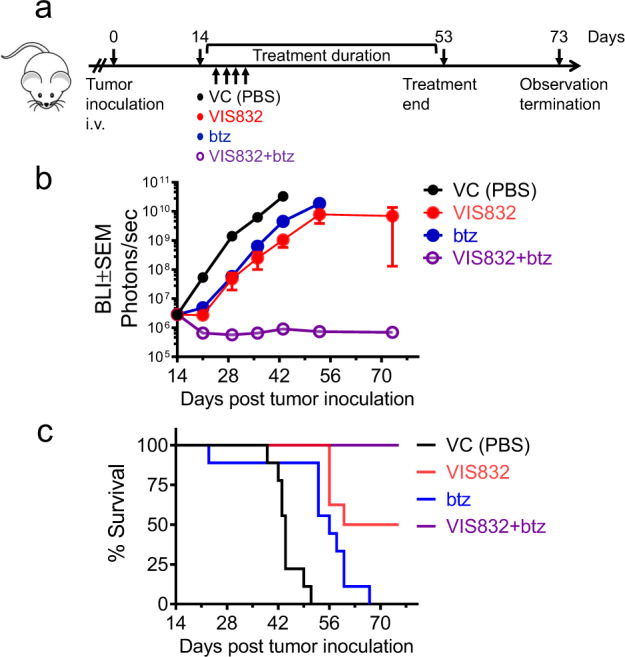
VIS832 treatment efficacy as monotherapy and in combination with btz in a murine model of disseminated human MM. **a** In vivo work plan. CB-17 SCID mice injected with MM1S-luc cells by i.v. route at day 0 were randomized on day 14 as the first day of dosing twice weekly with vehicle control (VC, PBS), VIS832 (4 mg/kg), btz (1 mg/kg), or combination of VIS832 and btz (*n* = 9 mice per group). Tumor burden was assessed by whole body BLI and quantified by IVIS imaging. Treatment was discontinued at day 53 (dosing phase), followed by a three-week washout period with end of study at day 73. **b** Group efficacy data. Disseminated tumor burden was reported as BLI ± SEM at each time point. Tumor growth was significantly inhibited starting at day 36 in VIS832 or btz vs. VC groups; the combination-treated vs. VC groups. (at Day 36, VIS832 vs. VC, *P* < 0.0001; btz vs. VC, *P* = 0.0001; VIS832 + btz vs. VC, *P* < 0.0001; VIS832 vs. VIS832 + btz, *P* < 0.0001; btz vs. VIS832 + btz, *P* < 0.0001; VIS832 vs. btz, *P* = 0.0004, by Tukey’s multiple comparison test). **c** Kaplan–Meier survival curves. The percentage of survival was defined as mice surviving to a pre-determined euthanasia criterion related to disease-related morbidity such as weight loss >20%, severely impaired central nervous system function, severely impaired movement, or loss of righting reflexes. VIS832 significantly increased the median overall survival of animals, and VIS832 with btz completely eradicated MM tumors, with all mice tumor-free (VC, 30d; VIS832 > 60d; btz, 42d; VIS832 + btz, >73d) (VIS832 vs. VC, *P* < 0.0001; btz vs. VC, *P* = 0.0004; VIS832 + btz vs. VC, *P* < 0.0001; VIS832 vs. VIS832 + btz, *P* < 0.02; btz vs. VIS832 + btz, *P* < 0.0001; VIS832 vs. btz, *P* = 0.02).

In vivo VIS832 efficacy was assessed using a murine model xenografted with the dissemination of MM1S-luc cells by i.v. injection into CB-17 SCID mice, which preserve aspects of innate immunity relevant to antibody mechanisms of action^29,31,32^, including NK cell- and macrophage-mediated cytotoxicity of antibody-targeted CD138-expressing MM cells. Dosing intervals and route of administration (*n* = 9 mice per group) were as described in methods and in Fig. 6a. Whole BLI of MM1S-luc cells was used to non-invasively quantify site-specific disease burden (Supplementary Fig. S7).

In the vehicle control (VC, PBS) group, the median time to evaluation size was ~28 days, and the median tumor burden doubling time was 2 days (range of 1.5–2.2 days), consistent with tumor implantation in all animals and an aggressive progression of tumor growth. The median survival of animals in the VC group was 30 days, and all animals were deceased by day 51 (Table 1 and Supplementary Tables S3 and 4). In contrast, VIS832 monotherapy at 4 mg/kg effectively reduced tumor burden with a prolonged time to evaluation (Supplementary Table S4, median survival of >60 days), and reduction of disseminated tumor growth (Fig. 6b and Supplementary Fig. S7). Five of 9 animals (56%) remained on study for its duration (day 73). Significant survival benefit of VIS832 was noted at day 53 (*P* < 0.0001, Fig. 6c), 3 weeks following treatment discontinuation. VIS832 treatment also led to disease regression in a subset of mice evaluated up to day 53 (the last day of dosing).

**Table 1:** Group (nine mice each group) summary for in vivo efficacy study.

Gp	Treatment	Dose (mg/kg)	Dose schedule	Max % weight change	% Treatment-related deaths	Efficacy			Survival		
						Tumor growth delay (days)	Median % Δ*T*/*C* (day 36)	%CR^e^	%TFS^f^	Median lifespan (days)^c^	% Increased lifespan
1	Vehicle	0.2 ml/20 g	5QW^a^	–13.6	--	--	--	--	--	30	--
2	VIS832	4.0	2QW	–4.3^d^	0.0	13.9	1.1	22.2		>60	>100
3	btz	1.0^b^	2QW	–13.6	11.1	10.6	8.2	0		42	40
4	VIS832+	1.0^b^	2QW	–9.8	0.0	>45	0.0	100	>90	>60	>100
	btz	4.0									

^a^Vehicle (PBS) administered by oral gavage.

^b^Dose level of bortezomib adjusted down from 2 mg/kg (2QW) to 1 mg/kg on day 18 due to toxicity including the death of one animal in treatment group (Gp).

^c^Measured from the day of first treatment in the study (not the day of tumor implant) for each animal. It captures the day of death for all animals that either die or are euthanized for disease or treatment-related causes. Animals euthanized for sampling or other reasons unrelated to disease or therapy (e.g., end of study) are excluded from this calculation.

^d^Negative change in weight observed during initial dosing only (see Fig. 6d).

^e^Complete regression (CR) (see Supplementary Tables S3 and 4).

^f^Tumor-free survivor (TFS) (see Supplementary Tables S3 and 4).

In the combination group, a sub-optimal dose of btz was chosen (1 mg/kg) that achieved a tumor growth delay of ~10 days and a 40% increase in lifespan, but no animals exhibited either partial or complete regression or survived (median survival of 42 days) to the end of study at Day 73 (Fig. 6 and Supplementary Table S4). VIS832 alone significantly extended host survival when compared with btz (*P* = 0.02). Combined VIS832 and btz treatments significantly enhanced efficacy, evidenced by complete tumor regression and 100% survival to end of study (Fig. 6c). Clear survival advantages were confirmed (*P* < 0.0001 for combination vs. VC; *P* < 0.02 for combination vs. VIS832; *P* < 0.0001 for combination vs. btz). This efficacy was sustained even after discontinuation of treatment on Day 53, 3 weeks prior to study termination. The apparent lack of any discernible tumor burden suggested an achievement of minimal residual disease in animals receiving combination therapy. Loss of body weight during the first week of treatment and mean body weight change was minimal (<5%). In fact, animals treated with 4 mg/kg VIS832 experienced an upward trend in mean body weight. In contrast, treatment-related toxicity was observed in the two groups initially receiving btz at the 2 mg/kg dose level, but reduction in dose from 2 mg/kg to 1 mg/kg led to recovery of body weight, particularly in mice receiving 1 mg/kg of btz and 4 mg/kg VIS832 combination.

Thus, VIS832 induced significant in vivo anti-MM activity as monotherapy, and further completely eradicated MM in mice treated in combination with btz.

## Discussion

Given its enhanced and prevalent presentation on the MM cell surface, CD138 is a compelling target for immunotherapy in MM. Many of the previously characterized anti-CD138 mAbs, such as BB4 in indatuximab ravtansine, target the integrin binding domain (IBD), an immunodominant region of CD138, which is distally located from the cell membrane. VIS832 evaluation has been sufficiently characterized with respect to its physicochemical and biophysical attributes, as measured by several different drug “developability” assays and metrics indicating favorable pharmaceutical properties. It engages a differentiated epitope on CD138 comprising two, non-contiguous regions that are required for optimal target binding. Importantly, when measured in the same assay, we showed stronger and more intense binding of VIS832 vs. BB4 to all MM cell lines, including those with relatively lower CD138 density. In parallel, further structural modeling of ADCC for well characterized antibodies such as rituximab with potent Fc-mediated ADCC indicates the critical importance of epitope location and topological proximity to the cell surface as a key determinant of immune synapse formation^46^. The epitope recognized by VIS832 includes a membrane proximal region of CD138 whose targeted binding is predicted to confer productive immune synapse formation for improved Fc effector dependent, immune cell-mediated cytotoxicity. As such, indatuximab ravtansine, which targets the IBD of CD138, is predicted to exhibit poor immune cell-based killing^25^ when compared with VIS832. Indeed, VIS832 induces superior MM cell lysis than the *BB4* possessing an isotype matched human IgG1.

VIS832-dependent NK cell-mediated cytotoxicity, while target dependent, did not precisely correlate with CD138 surface expression levels on target cells. This suggests a sufficiency of CD138 target abundance (threshold) to drive a potent antibody-mediated biological response. Other aspects include VIS832 target epitope engagement, target binding avidity, and other yet identified mechanisms of action. Significantly, our data indicate that the differentiated epitope of VIS832 in comparison with BB4 within indatuximab ravtansine confers a more productive target binding and augmented Fc effector function, leading to improved immune synapse formation, and ultimately to its more potent MM targeting and superior cellular cytotoxicity activity, including ADCC and ADCP.

Besides ADCC that presumptively represents a primary mechanism for the biological potency of VIS832, VIS832 delivered additional Fc-mediated ADCP to eliminate MM cells. ADCP activity of therapeutic mAbs including dara and GSK2857916 (belantamab mafodotin) is an important mechanism for their in vivo potency^29,35,41^. Significantly, VIS832 exhibited increased ADCP activity than dara in multiple MM cell lines, regardless of resistance to dex and IMiDs. VIS832 induced comparable ADCP to deplete RPMI8226-DR cells resistant to dara, indicating ADCP as a crucial mechanism of action of VIS832 to overcome dara resistance. This supplementary cytotoxic function of VIS832 confirms its multi-faceted immune-mediated anti-MM activity, which could aid in overcoming multi-drug resistance and prolonging response.

As in the case for dara, elotuzumab, isatuximab, and belantamab mafodotin^29,34,47–49^, the activating effect of len on immune effector cells also augments VIS832-induced cytotoxicity against MM cells. Such enhanced efficacy of combination vs. monotherapy likewise would be anticipated with other IMiDs including pom. Higher maximal killing of MM cells of VIS832 than dara also correlated with higher CD138 than CD38 target expression. VIS832 still potently induced ADCC against MM cells resistant to dara, either due to the loss of CD38, or acquired drug resistance through long-term culture selection with dara in ex vivo NK-MM co-cultures. Moreover, dara, but not VIS832, induced apoptosis in NK cells. Thus, VIS832 would not deplete NK cells, thereby avoiding any negative impact on its efficacy and therapeutic window.

VIS832, like its predecessor mAb 2810, induced robust immune cell-mediated cellular killing of patients MM cells resistant to btz, IMiDs, and/or dara. Its selective cytotoxicity against autologous patient MM cells was confirmed using whole BMMCs, as well as purified CD138 + cells freshly harvested from patients. Since various BM accessory cells confer immunosuppression, these data suggest that VIS832 would be active in the patient BM milieu. Its selective ADCC against autologous patient MM cells, but not CD138-negative BM cells, further supporting a favorable therapeutic index.

The in vivo efficacy of VIS832 was convincingly demonstrated in an aggressive disseminated MM1S xenograft model of human MM in CB-17 SCID mice, both as monotherapy and in combination with btz. When used alone, VIS832 reduced median tumor burden and improved overall survival. This efficacy was sustained after discontinuation of treatment 3 weeks prior to study termination. An evaluation of VIS832 PK/PD relationships in a prior dose range finding study in the same in vivo model indicated sufficient exposure of VIS832 at this dose level (4 mg/kg) to achieve a biological response (data not shown). Even lower dose levels, while not evaluated, are predicted to be sufficient to achieve such a response. Importantly, administration of VIS832 was well tolerated, with no overt treatment-related morbidities or toxicities, especially seen in btz-treated group.

Combined treatment of tumor-bearing mice with both VIS832 and dose-adjusted btz resulted in complete tumor regression and 100% survival of all animals to the end of study at day 73. Complete tumor elimination in all mice was sustained even after discontinuation of treatment on Day 53, 3 weeks prior to study termination. The apparent lack of any discernible tumor burden suggests an achievement of minimal residual disease in animals receiving the two therapies at sub-optimal doses in combination. Moreover, the superior efficacy of combined VIS832 and btz when compared to either agent alone suggests synergistic in vivo activity, consistent with synergistic in vitro cytotoxicity of this combination (CI < 1). Recently, CD138 downregulation sensitizes MM cells to btz treatment in another animal model^50^. The augmentation of activity of low-dose btz by VIS832 shown here suggests enhanced efficacy and tolerability at lower doses in the clinic.

Taken together, the significant in vivo efficacy of VIS832 in the MM1S xenograft murine model, coupled with its mechanisms of action and in vitro MM cytotoxicity, both confirm CD138 as a promising MM target and provide the basis for clinical development of VIS832 as a potentially effective mAb-based immunotherapy. Given the current therapeutic landscape and clinical approaches to MM treatment, the use of immune targeted therapies including VIS832 will likely be best suited to augment standard-of-care through an appropriate use of drug combinations, and/or as additional lines of therapies in MM. Such an approach will be of particular therapeutic value in the treatment of patients with relapsed disease and/or refractory to prior treatment (e.g., dara). Indeed, our current data indicate that VIS832 can overcome resistance to dex, btz, and IMiDs. Once its efficacy is established in RRMM, its favorable therapeutic index should allow for moving rapidly to earlier stages of disease, newly diagnosed MM and even SMM.

## Supplementary information

Supplementary materials
