# Supplementary material for: VIS832, a novel CD138-targeting monoclonal antibody, potently induces killing of human multiple myeloma and further synergizes with IMiDs or bortezomib in vitro and in vivo
Source: Blood Cancer J. 2020 Nov 2;10(11):110. doi: 10.1038/s41408-020-00378-z (PMC7643177; doi:10.1038/s41408-020-00378-z)
Supplement: Supplementary file 1 — Supplementary materials [file 41408_2020_378_MOESM1_ESM.pdf]

VIS832, a novel CD138-targeting monoclonal antibody, potently induces killing of human multiple myeloma and further synergizes with IMiDs or bortezomib *in vitro* and *in vivo*

Tengteng Yu[1,2], Bharat Chaganty[3], Liang Lin[1], Lijie Xing[1], Boopathy Ramakrishnan[3], Kenneth Wen[1], Phillip A Hsieh[1], Andrew Wollacott[3], Karthik Viswanathan[3], Hedy Adari[3], Shih-Feng Cho[1,4,5], Yuyin Li[1,6], Hailin Chen[1], Wenjuan Yang[1], Yan Xu[1,2], Gang An[2], Lugui Qiu[2], Nikhil Munshi,[1] Gregory Babcock[3], Zachary Shriver[3], James R. Myette[3], Kenneth C Anderson,[1]* Yu-Tzu Tai[1]*

[1] Jerome Lipper Multiple Myeloma Center, LeBow Institute for Myeloma Therapeutics, Dana-Farber Cancer Institute, Harvard Medical School, Boston MA, USA
[2] State Key Laboratory of Experimental Hematology, Institute of Hematology and Blood Diseases Hospital, Chinese Academy of Medical Science and Peking Union Medical College, Tianjin, China.
[3] Visterra Inc., Waltham, MA
[4] Division of Hematology & Oncology, Department of Internal Medicine, Kaohsiung Medical University Hospital, Kaohsiung Medical University, Kaohsiung 80708, Taiwan
[5] Faculty of Medicine, College of Medicine, Kaohsiung Medical University, Kaohsiung 80708, Taiwan
[6] School of Biotechnology, Tianjin University of Science and Technology, Key Lab of Industrial Fermentation Microbiology of the Ministry of Education, State Key Laboratory of Food Nutrition and Safety, Tianjin 300457

*To whom correspondence should be addressed: Yu-Tzu Tai, Ph.D., Department of Medical Oncology, Dana-Farber Cancer Institute, M551, 450 Brookline Avenue, Boston, MA 02215. Phone: (617) 632-3875; Fax: (617) 632-2140; E-mail: yu-tzu_tai@dfci.harvard.edu; Kenneth C Anderson, M.D., Department of Medical Oncology, Dana-Farber Cancer Institute, M557, 450 Brookline Avenue, Boston, MA 02215 Phone : (617) 632-2144 ; Fax : (617) 632-2140 ; Email : kenneth_anderson@dfci.harvard.edu

Supplemental methods

Production of VIS832

VIS832 was produced by the transient co-transfection of two mammalian expression vectors separately encoding heavy chain (HC) and light chains (LC) for production as full length, human IgG1kappa antibodies in Chinese hamster ovary cells (ExpiCHO, Thermo Fisher Scientific). DNA transfection was performed using lipid-based transfection reagents (e.g., ExpiFectamine CHO Transfection Kit, Thermo Fisher Scientific) and standard protocols as recommended by the manufacturer. VIS832 was produced under cell culture conditions designed to reduce core fucose in the respective N-glycans at asparagine 297 and ultimately for the purpose of further enhancing Fc-mediated ADCC activity. Highly-reduced core fucosylation was achieved metabolically through the use of the decoy substrate 2-deoxy-2-fluoro-l-fucose (2FF), which was added concurrently to the culture media at 0.15 mM at four hours following transient transfection. Cell culture was carried out in shake flasks at 600 ml cell culture scale for a period of 7 days without feed supplementation. Secreted monoclonal antibodies were subsequently purified from cell culture media using protein A affinity capture on Fast Protein Liquid chromatography (FPLC). Purified VIS832 was formulated in phosphate buffered saline (PBS), pH 7.4, and quantified by UV absorbance at 280 nm using a theoretically derived extinction coefficient of 1.41 ($\varepsilon_{0.1\%}$). Purity and molecular mass were confirmed by both SDS-PAGE and SEC-HPLC, validating predicted molecular weight and >95% purity.

Reagents (Test antibodies)

BB4 - The mouse anti-CD138 antibody clone BB4 was cloned as a chimeric molecule with human IgG1 heavy chain constant region (Fc), recombinantly produced by recombinant transfection in EXPI 293 as an afucosylatated antibody and purified by Protein A affinity capture chromatography, as otherwise described for VIS832.

Daratumumab - Darzalex® (Daratumumab), 5 mg/20 ml solution, was purchased through Pharmaceutical Buyers International, a fully licensed third-party purchaser of pharmaceutical products for use in research.

MM cell culture and patient derived cells

Unless otherwise stated, all human MM cell lines express CD138 and BCMA, and they were obtained from American Type Culture Collection (ATCC), as previously described[1-3]. Drug resistant cell lines including H929 cells resistant to len/pom H929(R) and MM1S resistant to len/pom MMIS(R) were generated by culturing these cell lines in medium containing gradually increasing concentrations of len and pom (up to 100 µM)[1]. CRISPR/CAS9 technique was used to knock out CD138 expression in H929 and JJN3 cells. Similar technique was used to knock out CD38 expression in H929 cells to generate H929 CD38 KO-1 and H929 CD38 KO-2. All MM cell lines were cultured in media containing RPMI-1640 (Invitrogen, Carlsbad, CA), 10% FBS (Hyclone, Logan, UT), HEPES buffer (ThermoFisher Scientific), L-glutamine (ThermoFisher Scientific), penicillin, and streptomycin (ThermoFisher Scientific). They were routinely checked by Human short tandem repeat (STR) DNA Profiling Cell Authentication for their authenticity and for mycoplasma contamination by PCR and the MycoSEQ Mycoplasma detection assay kit (ThermoFisher Scientific), as previously described[1,2]. MM1S, MM1S(R), and H929(R) cell lines were subject to the generation of stable luciferase reporter lines (MM1S-luc,

MM1S(R)-luc, H929(R)-luc) for the use in indicated ADCC assays. MM1S-luc cells were also used for *in vivo* work.

Healthy donors and MM patient samples were obtained with informed consent, in accordance with the Declaration of Helsinki and under the auspices of a Dana-Farber Cancer Institute Institutional Review Board (IRB) approved protocol. Primary CD138+ plasma cells were purified from BM aspirates using anti-CD138 microbeads (Miltenyi Biotech, Auburn, CA). Residual CD138-negative BM mononuclear cells (BMMCs) were further cultured for 3 to 6 weeks to derive stromal cells (BMSCs).

Natural Killer (NK) cells were collected from fresh buffy coat of healthy donors with the RosetteSep human NK cell enrichment cocktail (StemCell Technologies Inc. Vancouver, Canada). Peripheral blood mononuclear cells (PBMCs) were obtained from healthy donors or MM patients. Monocytes were purified from PBMCs using anti-CD14 microbeads (Miltenyi Biotech, Auburn, CA). CD14+ monocytes were further cultured with macrophage colony-stimulating factor (M-CSF, 150 ng/ml) (PeproTech, Rocky Hill, NJ) for macrophage (CD11b+) differentiation.

Flow cytometric analysis

Cell surface CD138 expression was quantified by flow cytometry using an PE-anti-human CD138 antibody clone DL-101 (BioLegend) analyzed on a BD Fortessa flow cytometer (BD Biosciences, San Jose, CA). Data were analyzed using Flowjo version 10 (Tree Star, Inc., Ashland, OR).

Anti-CD138 monoclonal antibodies VIS832 and BB4 were also assessed by flow cytometry for their relative capacity to bind to CD138 on the surface of CD138+ human myeloma cell lines ($n \geq 8$). MM cells were cultured with recombinant antibody at varying concentrations as noted for up to 4 hours followed by washing in PBS and detection using secondary anti-human IgG1 antibody conjugated with APC or related fluorophore. Antibody cell binding was reported as geometric mean fluorescence intensity (MFI).

Real-time quantitative RT-PCR (qRT-PCR)

Total RNA was isolated using the TRIzol reagent (Life Technologies), according to manufacturer's instructions. cDNA was obtained from 0.5 µg of RNA using the cDNA Reverse Transcription kit (Life Technologies). Real-time PCR was performed using TaqMan gene expression assay primer sets for SDC1 (CD138) (Hs00896423_m1), CD38 (Hs01120071_m1) and internal controls (18S, HS03003631_g1; GAPDH, Hs02758991_g1) on an ABI Prism 7700 sequence detection system (Applied Biosystems).

ADCC Reporter Assay

VIS832 and other anti-CD138 antibodies were independently evaluated for antibody dependent cellular cytotoxicity (ADCC) activity against U266 target MM cell line using an orthogonal luciferase based ADCC reporter bioassay (Catalog number G7010, Promega). In this assay, engineered Jurkat cells were stably transfected with the human FcγRIIIa receptor and served as surrogate effector cell line. U266 target cells were incubated with mAbs that were 3-fold serially diluted from a starting concentration of 3 µg/ml and subsequently added to effector cells at a cell ratio of effector: target (E:T) of 10:1. After 16-hour incubation at $37^{o}C$, luciferase activity was assessed as a measure of ADCC activity and reported as relative luminescence units (RLU). ADCC data were plotted using nonlinear regression analysis and a 4-parameter curve fit.

Controls include effector cell only to determine non-specific background. Data are typically plotted as fold-induction of ADCC activity relative to no antibody control.

Syndecan-1 ELISA

Soluble syndecan-1 levels in cell culture media were quantified by a sandwich enzyme-linked immunosorbent assay (ELISA) using Human Syndecan-1 DuoSet ELISA Kit (R&D Systems, Inc., MN). The syndecan-1 concentrations in the samples were determined by a standard protein curve, according to the manufacturer's protocol. The reported assay range is listed by the vendor as 125.0 - 8,000 pg/ml. The same assay was also used to quantify syndecan-1 in cell culture supernatants.

Luciferase-based ADCC assay

In experiments comparing the ADCC activities of VIS832 and daratumumab, a modified luciferase-based assay was also used. In brief, MM1S-, MM1S(R)-, and H929(R)-luc cell lines were used as target cells to co-culture with human NK cells at an E:T ratio of 10:1. Both target and effector cells were also cultured in the presence of varying concentrations of either VIS832 or daratumumab for 4 hours. Cells were collected by centrifugation and lysed with 20 µl passive lysis buffer (to establish 100% lysis); firefly luciferase value was quantified in accordance with instructions as provided in the Dual-Luciferase® Reporter Assay System Technical Manual.

VIS832-induced cytotoxicity of patient derived myeloma cells

The potency of VIS832 in mediating ADCC against patient derived cells was evaluated in two separate methods. In the first method, BMMCs derived from RRMM patients were used as both the source of target and effector cells. These cells were directly treated with varying concentrations of VIS832 (0-10 µg/ml) for 24 hours. The population of patient derived plasma cells was detected and quantified by flow cytometry using BCMA as a surrogate plasma cell surface marker. The % of viable $BCMA^+$ patient MM cells remaining was used to determine the extent of cell killing ability of VIS832.

In the second method, MM cells from refractory/relapsed patients were sorted by CD138 microbeads. CD138+ or CD138- cells were co-cultured for 1.5 hours with PBMCs from the matched individual (E:T = 10:1), in the presence of varying concentrations of VIS832 (0-1 µg/ml). Target cells (CD138+ or the matched CD138-cells) were pre-labeled with Calcein AM, followed by the Calcein AM release assay to determine CD138-specific cytotoxicity (% lysis) of VIS832.

Combined use of VIS832 and lenalidomide or bortezomib

ADCC activity was measured as described above using NK cells and calcein AM-pre-labeled MM cell lines ($n\geq4$), with some modifications. Target MM cell lines were co-treated with sub-optimal doses of len or btz (Selleck Chemicals) and varying concentrations of VIS832 in the presence of effector cells (lower E:T ratio of 4:1) for a period of 2 hours, prior to measuring released calcein-AM. In some experiments, various MM cell lines were pre-treated with len or btz for 10 hours prior to VIS832-induced ADCC assays (E:T ratio of 1:1) for 1.5 hours prior to analysis of cellular lysis.

Murine model of disseminated human MM disease

Five-to-seven-week-old female CB-17 SCID mice (C.B-17/IcrHsd-*Prkdc^{scid}*) were injected intravenously (i.v.) with 5.0×10^6 live (trypan-excluding) MM1S-luc cells on day 0 and staged for therapeutic dosing on day 14, after normalizing the baseline tumor burden between the groups set by bioluminescence (BLI) as 2.90×10^6 photons/second (p/s, range of group means, $2.81\text{-}3.07 \times 10^6$ p/s). Whole body tumor burden was monitored by BLI using an IVIS Lumina S5 (PerkinElmer, MA). At 14 days after tumor implant, mice (n=9 per group) were randomized to ensure that the mean whole-body tumor burden for all groups was within 10% of the overall mean whole-body tumor burden for the study population before treatments started. Animals were imaged five at a time under 1-2% isoflurane gas anesthesia. Each mouse was injected IP with 150 mg/kg (15 mg/ml) D-luciferin (Promega) formulated in saline for injection and imaged in the prone, then supine positions 10 minutes after the injection. Large binning of the CCD chip was used, and the exposure time was adjusted (5 seconds to 2 minutes) to obtain at least several hundred counts per image and to avoid saturation of the CCD chip.

Animals were imaged once weekly at staging and on days 14, 21, 29, 36, 43, 53, and 73 (end of study) for all animals remaining on study. Images were analyzed using Living Image 4.7.1 (PerkinElmer, MA) software. Whole body fixed-volume regions of interest (ROIs) were placed on prone and supine images for each individual animal and labeled based on animal identification. Total flux (photons/sec) was calculated and exported for all ROIs to facilitate analyses between groups. The prone and supine ROIs were summed together to estimate whole body tumor burden. Subject ROIs were also placed over each mouse, and region-specific ROIs were placed over spine and hindlimbs for each animal. These ROIs were then quantified independent of the whole-body ROIs in order to calculate signal from only hind limbs or spine.

Primary and secondary efficacy were generated using *in vivo* whole body BLI and included tumor burden as defined. Endpoints included whole body BLI ratio T/C (where T = treatment group and C = vehicle control) measured on each imaging day and reported on day 36, and tumor growth delay, as measured as time to tumor burden as pre-defined as a BLI signal of 1.0×10^9 p/s. Additional efficacy endpoints included tumor regression, tumor free survival (TFS), and median life span normalized to vehicle control (% increased life span); this latter endpoint was calculated based on the first day of treatment and not the day of implant. Overall survival was calculated based on animals remaining on study.

Additional measurements included body weights (3x weekly), daily clinical observations, and necropsy following euthanasia. Pre-defined euthanasia criteria were related to disease progression and included >20% loss in body weight or disease-related morbidity such as hind limb paralysis, paresthesia, or other signs of severe CNS or musculoskeletal impairment.

Statistical analysis

Multiple groups (≥3) were analyzed by one-way ANOVA, and paired groups were analyzed by two-way ANOVA or Student t test. All data were graphed and analyzed using GraphPad Prism 8.3.1 (San Diego, CA) with $P<.05$ indicating statistical significance. Tumor growth curves *in vivo* were analyzed by Tukey's multiple comparison test and 2-way ANOVA, after log-transformation of data. The survival curve in the murine model was plotted by using the Kaplan-Meier method, and compared by the log-rank test.

Drug interactions between VIS832 and len or btz were assessed by CompuSyn software using the Chou-Talalay method to determine combination index (CI)[4]. CI<1 indicates synergism whereas CI>1 antagonism and CI=1 additive effect.

Reference

1. Xing L, Lin L, Yu T, et al. A novel BCMA PBD-ADC with ATM/ATR/WEE1 inhibitors or bortezomib induce synergistic lethality in multiple myeloma. *Leukemia.* **34**, 2150-2162 (2020).

2. Tai YT, Chang BY, Kong SY, et al. Bruton tyrosine kinase inhibition is a novel therapeutic strategy targeting tumor in the bone marrow microenvironment in multiple myeloma. *Blood.* **120**, 1877-1887 (2012).

3. Tai YT, Landesman Y, Acharya C, et al. CRM1 inhibition induces tumor cell cytotoxicity and impairs osteoclastogenesis in multiple myeloma: molecular mechanisms and therapeutic implications. *Leukemia.* **28**, 155-165 (2014).

4. Chou TC. Drug combination studies and their synergy quantification using the Chou-Talalay method. *Cancer Res.* **70**, 440-446 (2010).

Table S1. Fold increases in binding affinity of VIS832 vs. BB4 to CD138 on MM cell membrane.

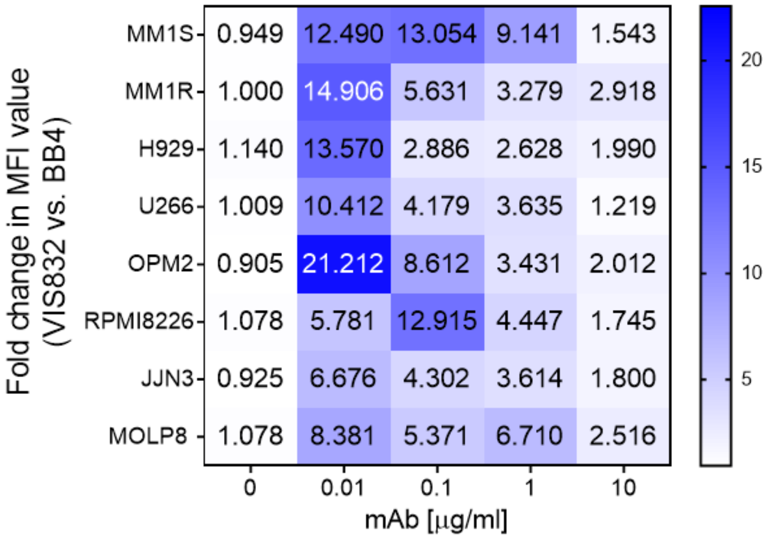

Table S1

Table S1. Fold increases in binding affinity of VIS832 vs. BB4 to CD138 on MM cell membrane.

Target binding of VIS832 vs BB4 mAb in MM cell lines was evaluated by quantitative flow cytometry (FC) analysis using an APC-conjugated anti-human IgG1 antibody for detection (**Fig. 1A**). Binding intensity was reported as geometric mean fluorescence intensity (MFI) normalized to isotype control. Isotype control for VIS832 and BB4 (10 µg/ml) were used as background controls (n=3 replicates; ± SEM). Fold changes were calculated by the MFI values for VIS832 divided by those for BB4.

Table S2. EC_{50} of VIS832 and its chimeric predecessor 2810 mAb

VIS832

MM cell line	Maximum lysis (%) ± SEM	EC_{50} (ng/ml) ± SEM	CD138 MFI ± SEM
MM1S	75.8±2.01	6.65±3.31	2076.92±16.05
MM1R	87.7±1.79	3.80±2.14	3889.1±15.24
H929	96.5±0.68	4.34±1.79	6641.21±20.63
U266	86.1±0.81	6.68±3.15	4594.13±17.08
OPM2	89.8±1.59	6.36±1.51	5507.98±14.04
RPMI8226	50.1±2.73	12.52±3.31	2298.17±14.15
JJN3	47.3±0.43	14.48±2.81	1640.55±22.67
MOLP8	40.2±0.13	12.11±0.31	2886.63±18.54
MM1S(R)	86.5±5.53	6.24±1.47	3684.39±17.22
H929(R)	97.3±3.34	2.22±0.37	6536.50±18.45
ANBL6	39.37±0.70	14.9±2.24	1564.5±17.30
ANBL6-BR	37.06±1.45	15.3±2.71	1468.0±18.30

2810 mAb

MM cell line	Maximum lysis (%) ± SEM	EC_{50} (ng/ml)
MM1S	79.9±1.01	8.85
MM1R	86.6±1.93	4.568
H929	79.7±1.74	4.75
U266	89.99±4.64	2.387
OPM2	78.82±3.78	6.65
RPMI8226	37.96±1.86	19.61
JJN3	42.76±2.64	27.94
MOLP8	39.64±4.88	28.16
MM1S(R)	78.0±5.91	20.42
H929(R)	60.36±3.34	2.29

The EC_{50} is the antibody concentration to achieve half-maximal lysis

Table S2

Table S2. EC_{50} of VIS832 and its chimeric predecessor 2810 mAb.
VIS832 (left)- and 2810 mAb (right)-induced ADCC to lyse indicated MM cell lines (n=12, left; n=10, right) using the calcein-AM release assay (**Fig. 2A**, Supplementary Figure S3A, C). Human NK effector cells from 3 healthy donors were incubated with indicated MM cell lines (E:T=10:1) in a serial concentration of VIS832. The percent maximum lysis and EC_{50} were determined from 3 independent experiments, with triplicate measurements for each assay. CD138 MFI values was determined by flow cytometry analysis using anti-CD138 mAb DL101 clone (Supplementary Figure S1A). Data are shown as means (% lysis) ± SEMs of three independent experiments.

Table S3. Summary of *in vivo* study endpoints.

All efficacy endpoints were generated using *in vivo* whole body BLI signals.

Data Type	Primary Endpoint 1	Primary Endpoint 2	Secondary Endpoints 1	Secondary Endpoint 2
**Pharmacology**	Treatment related deaths (%)	Treatment related weight change (%)	NA	NA
**Efficacy**	Median %T/C on Day 36	Tumor growth delay (days)	%CR, %PR, %TFS	Increased lifespan

Table S4. Group (9 mice each group) summary for *in vivo* efficacy study

Definitions for animal efficacy endpoints.

Median T/C – Is a group endpoint. It is calculated for each day of treatment as:

$$Median\frac{T}{C} = \left(\frac{\boldsymbol{Treatment}_{med}}{\boldsymbol{Control}_{med}}\right) * 100$$

Time to Evaluation Size (TES) – TES is an individual mouse endpoint, and it is expressed in days from tumor implant. It is the time it takes the tumor burden to reach a specified value, for this study, tumor burden is evaluated by BLI. It is calculated by log-linear interpolation between the two closest data points that bracket the chosen tumor burden.

$$D_{ES} = D_h - \frac{\left((\log V_h - \log ES) * (D_h - D_l)\right)}{(\log V_h - \log V_l)}$$

where:

$D_{ES} = TES_i$ - the day evaluation size is reached

D_h - the day of the first measurement greater than the ES was reached

D_l - the day of the last measurement before the ES was reached

V_h - The tumor burden on day D_h

V_l - the tumor burden on D_l

ES - the evaluation size

Tumor growth delay (TGD) – TGD is a group endpoint. Tumor growth delay is expressed in units of days and is calculated from the median times it takes the mice in a group to reach a specified tumor burden (time to evaluation size, TES as defined above). It can be calculated as:

TGD = median TES $_{\text{treated}}$ – median TES $_{\text{control}}$

Complete regression (CR)—An animal is credited with a complete regression if its tumor burden decreases to less than a declared background BLI signal level. In this particular study, this background signal level was measured as 7.20E+05p/s

Tumor-free Survivor (TFS) – A TFS is any animal that (1) survives until termination of the study, and (2) has no reliably measurable evidence of disease at study termination. Mice that are tumor–free at some point during the study but are then euthanized for sampling or other purposes prior to the end of the study are not considered in this assessment. They are excluded from calculation of the %TFS.

Median Lifespan – Lifespan is an individual mouse endpoint. It is measured from the day of first treatment in the study (not the day of tumor implant) for each animal. It captures the day of death for all animals that either die or are euthanized for disease or treatment related causes. Animals euthanized for sampling or other causes unrelated to disease or therapy are excluded from this calculation. The median lifespan for the group is used to calculate the % Increase in lifespan (%ILS). When animals are euthanized for IACUC mandated maximum tumor burdens, Time to Progression (TP) is used instead of this variable.

% Increase in lifespan (ILS) - %ILS is a group endpoint. It is calculated as:

$$\%ILS = \left\{\frac{[(median\ Treated\ Lifespan) - (median\ Control\ Lifespan)]}{median\ Control\ Lifespan}\right\} * 100$$

Supplementary Figure Legends

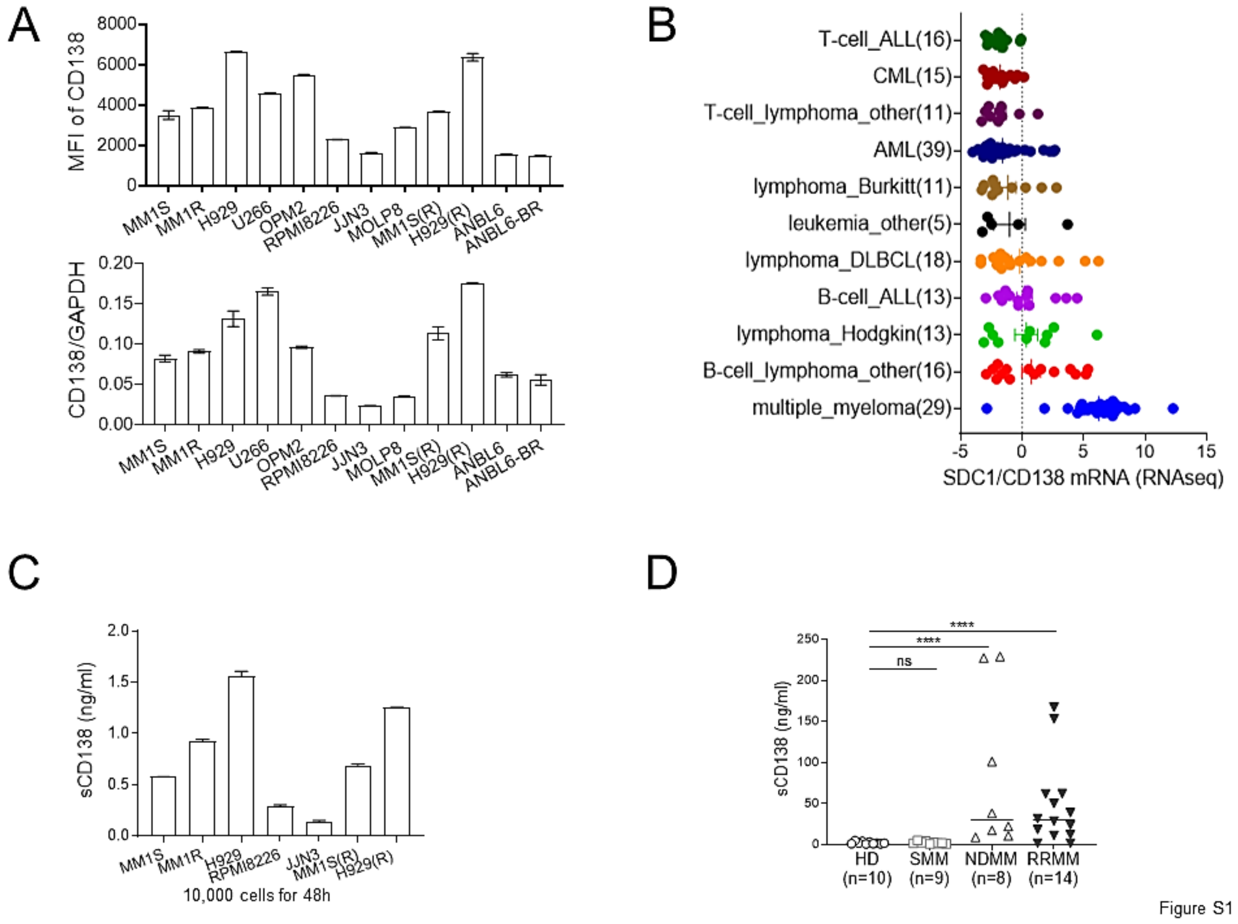

Figure S1. Relative protein and transcript levels of SDC1/CD138 in MM cells
A, CD138 expression was analyzed in MM cell lines by flow cytometry (upper panel, anti-CD138 mAb DL-101 clone) and real-time qRT-PCR (lower panel). Data represented the mean ± standard error of mean (SEM, error bar) of three independent experiments. **B**, Transcript levels were expressed as log2 changes as normalized to reference cell lines. Data derived from the Cancer Cell Line Encyclopedia (CCLE). SDC1/CD138 expression in MM (n=29) was significantly higher than that in other cell lines in hematological malignancies. http://www.broadinstitute.org/ccle. **C**, Quantification of soluble CD138 (sCD138) in cell cultured medium (CM) in MM cell lines by ELISA. Cells were cultured in 96 wells for 48h at a same concentration (10,000 cells/well). **D**, Soluble CD138 (sCD138) in serum samples of MM patients vs healthy donors was analyzed by ELISA. ns, not significant, ****P<.001.

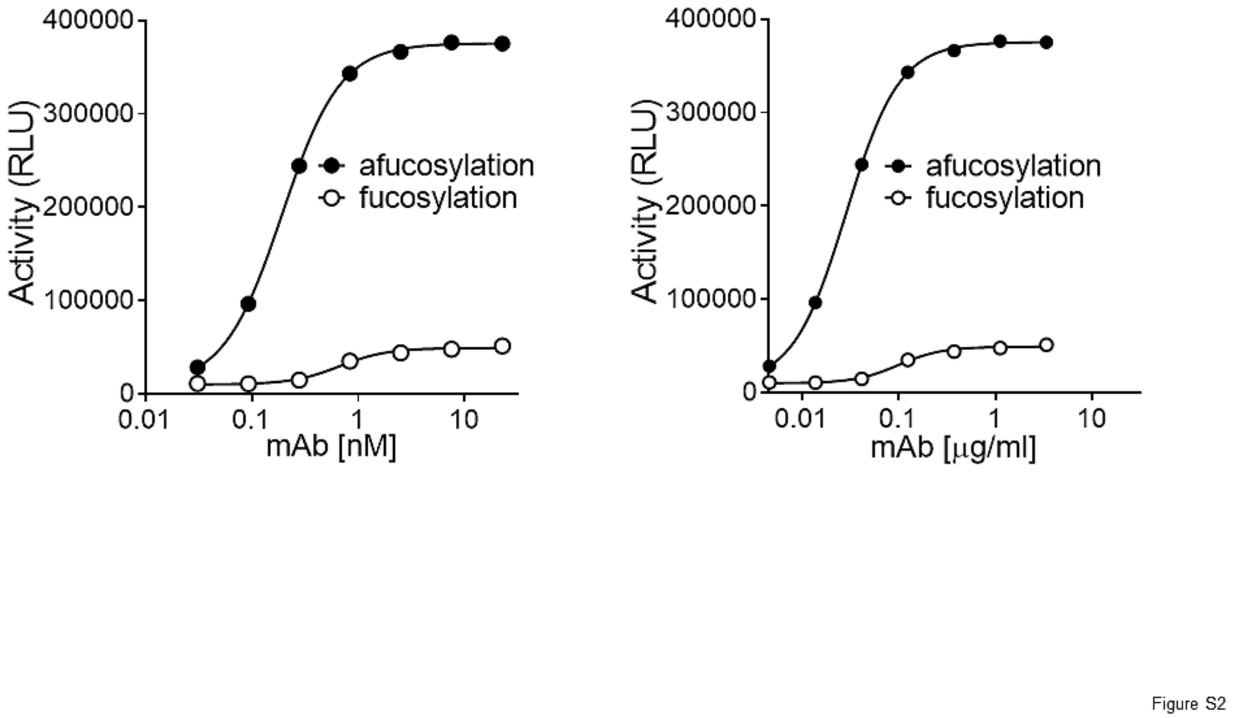

Figure S2. Optimization of VIS832

Fc afucosylation further increased maximal ADCC in the Promega reporter cell assay, where Jurkat cells stably transfected with human FcRIIIa as surrogate for effector cells were co-cultured with target U266 MM cell line (as in Figure 1C).

.

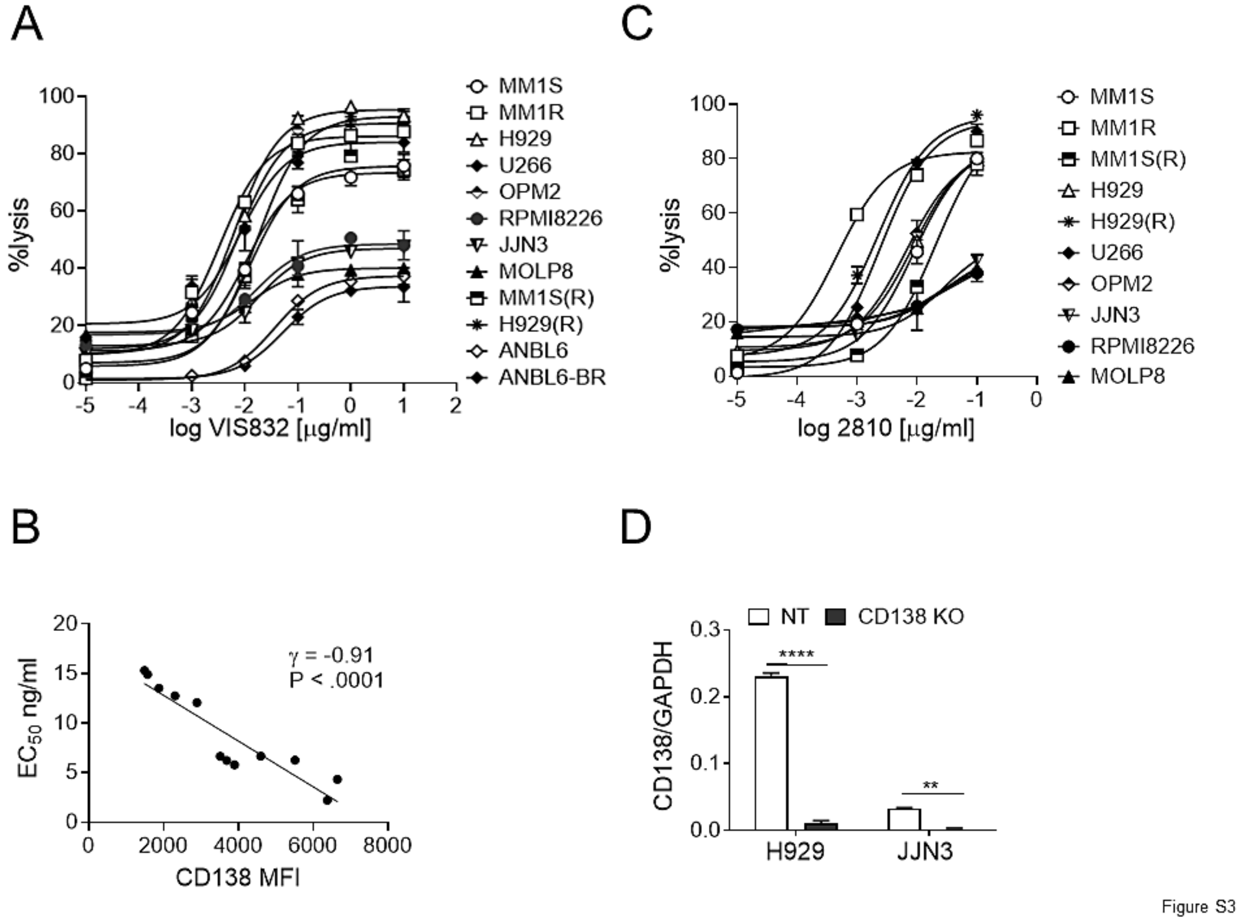

Figure S3. CD138-specific ADCC activity induced by VIS832 and its parental 2810 mAb in MM cell lines

A, Dose response curves for VIS832. **B**, The correlation analysis of CD138 MFI with the EC_{50} of VIS832. **C**, 2810 mAb (a mouse-human chimeric antibody as the predecessor for humanization of VIS832)-induced ADCC against 10 MM cell lines. **D**, CD138 gene KO was confirmed in H929 and JJN3 MM cell lines engineered using CRISPR/CAS9 gene editing. **P<.01, ****P<.001

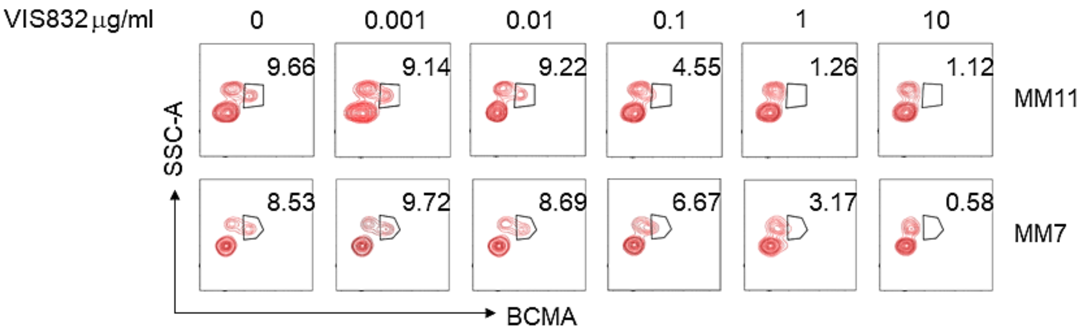

Figure S4

Figure S4. ADCC activity of anti-CD138 antibody VIS832 against myeloma cells from RRMM patients.
Example of flow cytometry gating strategy on VIS832 autologous killing in samples of patients with MM. VIS832 was incubated for 24 hours with autologous patient MM cells and effector immune cells isolated from bone marrow aspirates. Cell killing was assessed by flow cytometry to monitor antibody-dependent loss of BCMA+ plasma cells, as described for VIS832 in methods. More than 90% lysis of patient (BCMA+) MM cells was achieved in selected patients.

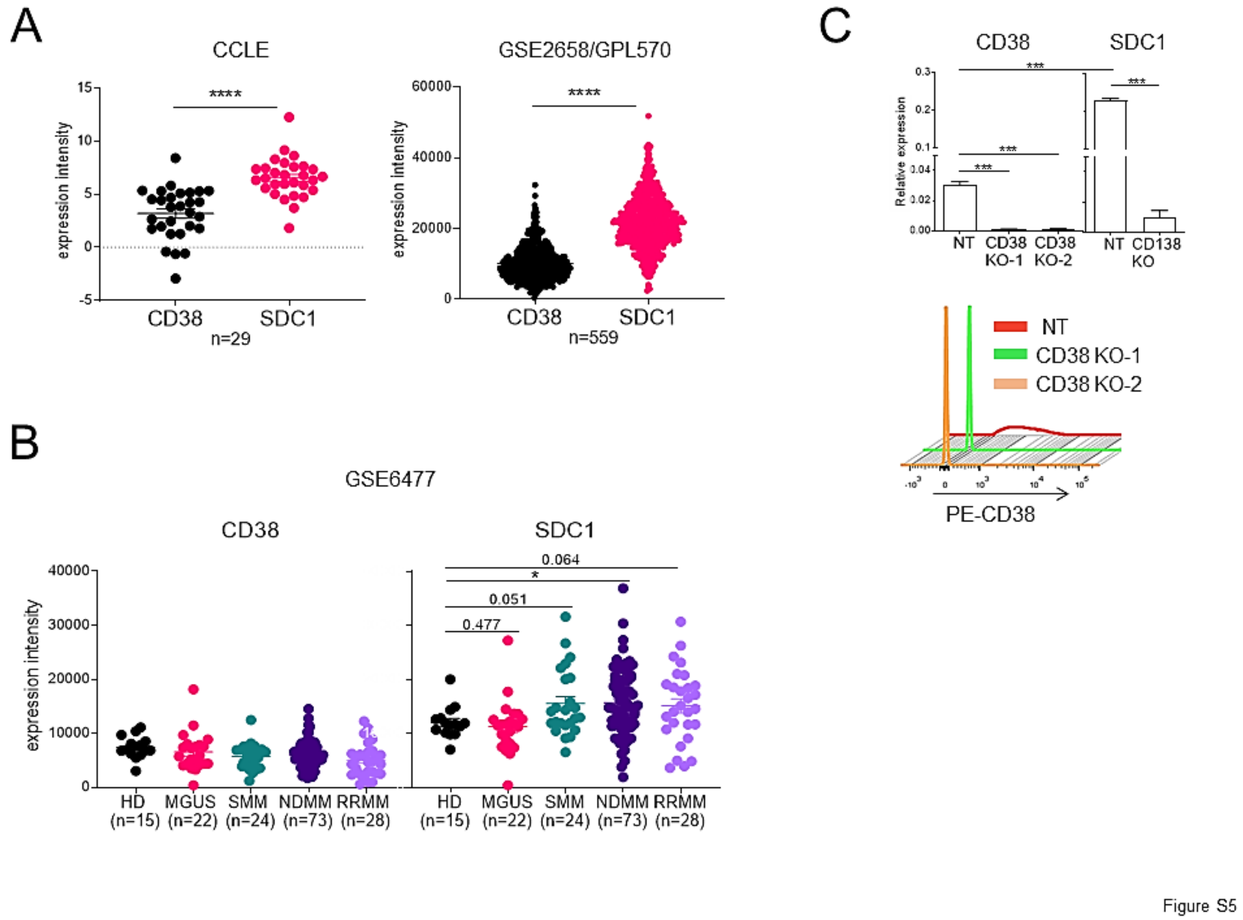

Figure S5. **CD138 (SDC1) vs CD38 expression in patient MM cells, and confirmation of CD38 KO in H929 transfectants.**
MM cell lines (n=29, CCLE database) including H929 (**A, C**) and patient MM cells (**A**, GSE2658; **B**, GSE6477) express significantly higher CD138 than CD38. Furthermore, CD138, but not CD38, was increased with progressive disease, as shown in GSE6477 (left), a published patient MM cell database. **C**, CD38 gene knockout in H929 cells engineered by CRISPR/CAS9 gene editing, real-time qRT-PCR (upper panel); and flow cytometry (lower panel) was performed for verification of CD38 KO. *P<.05, ****P<.0001

A Target cell: MM1S — No pre-treatment

VIS832 (μg/ml)	len (μM)	Fa	CI
0.001	1.0	0.16	0.08
0.01	1.0	0.25	0.08
0.1	1.0	0.34	0.14
1.0	1.0	0.43	0.34
0.001	5.0	0.17	0.06
0.01	5.0	0.30	0.03
0.1	5.0	0.39	0.06
1.0	5.0	0.49	0.13
0.001	10.0	0.20	0.02
0.01	10.0	0.33	0.02
0.1	10.0	0.54	0.006
1.0	10.0	0.57	0.04

MM1R

VIS832 (μg/ml)	len (μM)	Fa	CI
0.001	1.0	0.33	0.007
0.01	1.0	0.46	0.02
0.1	1.0	0.50	0.17
1.0	1.0	0.59	0.78
0.001	5.0	0.35	0.006
0.01	5.0	0.47	0.02
0.1	5.0	0.50	0.16
1.0	5.0	0.60	0.72
0.001	10.0	0.39	0.004
0.01	10.0	0.47	0.02
0.1	10.0	0.56	0.10
1.0	10.0	0.64	0.51

No pre-treatment

B Target cell: MM1S

VIS832 (μg/ml)	len (μM)	Fa	CI
0.001	1.0	0.39	0.02
0.01	1.0	0.49	0.009
0.1	1.0	0.56	0.008
1.0	1.0	0.59	0.02
0.001	5.0	0.46	0.03
0.01	5.0	0.53	0.02
0.1	5.0	0.85	0.002
1.0	5.0	0.95	2.99E-04
0.001	10.0	0.49	0.05
0.01	10.0	0.64	0.02
0.1	10.0	0.91	0.002
1.0	10.0	0.97	2.74E-04

MM1R

VIS832 (μg/ml)	len (μM)	Fa	CI
0.001	1.0	0.26	0.18
0.01	1.0	0.64	0.004
0.1	1.0	0.81	0.001
1.0	1.0	0.91	4.62E-04
0.001	5.0	0.33	0.05
0.01	5.0	0.71	0.002
0.1	5.0	0.87	2.24E-04
1.0	5.0	0.94	8.67E-05
0.001	10.0	0.38	0.02
0.01	10.0	0.77	3.18E-04
0.1	10.0	0.88	1.58E-04
1.0	10.0	0.95	4.15E-05

Target cell pre-treatment

Figure S6

C Target cell: RPMI8226

VIS832 (μg/ml)	btz (nM)	Fa	CI
0.001	1.0	0.1	0.11
0.01	1.0	0.16	0.04
0.1	1.0	0.17	0.09
1.0	1.0	0.32	0.01
0.001	2.0	0.11	0.15
0.01	2.0	0.11	0.27
0.1	2.0	0.35	0.008
1.0	2.0	0.52	0.002
0.001	4.0	0.13	0.19
0.01	4.0	0.24	0.05
0.1	4.0	0.35	0.02
1.0	4.0	0.55	0.003

H929

VIS832 (μg/ml)	btz (nM)	Fa	CI
0.001	1.0	0.18	0.20
0.01	1.0	0.22	0.18
0.1	1.0	0.37	0.11
1.0	1.0	0.39	0.12
0.001	2.0	0.20	0.36
0.01	2.0	0.32	0.25
0.1	2.0	0.41	0.20
1.0	2.0	0.60	0.13
0.001	4.0	0.25	0.61
0.01	4.0	0.33	0.49
0.1	4.0	0.52	0.32
1.0	4.0	0.62	0.26

No pre-treatment

D Target cell: RPMI8226

VIS832 (μg/ml)	btz (nM)	Fa	CI
0.001	1.0	0.18	0.22
0.01	1.0	0.21	0.17
0.1	1.0	0.22	0.16
1.0	1.0	0.23	0.15
0.001	2.0	0.2	0.38
0.01	2.0	0.22	0.32
0.1	2.0	0.33	0.16
1.0	2.0	0.38	0.12
0.001	4.0	0.24	0.56
0.01	4.0	0.34	0.30
0.1	4.0	0.41	0.20
1.0	4.0	0.64	0.06

H929

VIS832 (μg/ml)	btz (nM)	Fa	CI
0.001	1.0	0.18	0.17
0.01	1.0	0.26	0.09
0.1	1.0	0.42	0.04
1.0	1.0	0.49	0.04
0.001	2.0	0.18	0.32
0.01	2.0	0.29	0.14
0.1	2.0	0.46	0.05
1.0	2.0	0.55	0.04
0.001	4.0	0.37	0.17
0.01	4.0	0.49	0.09
0.1	4.0	0.67	0.03
1.0	4.0	0.70	0.03

Target cell pre-treatment

Figure S6

Figure S6. Raw data to derive CI values in Figure 4.
Combination index (CI) was determined by the Chou-Talalay method. CI<1.0 corresponds to a synergistic interaction. len: lenalidomide; btz: bortezomib**.** Indicated target MM cells were pre-treated without (**A**, **C**) or with len (**B**) or btz (**D**).

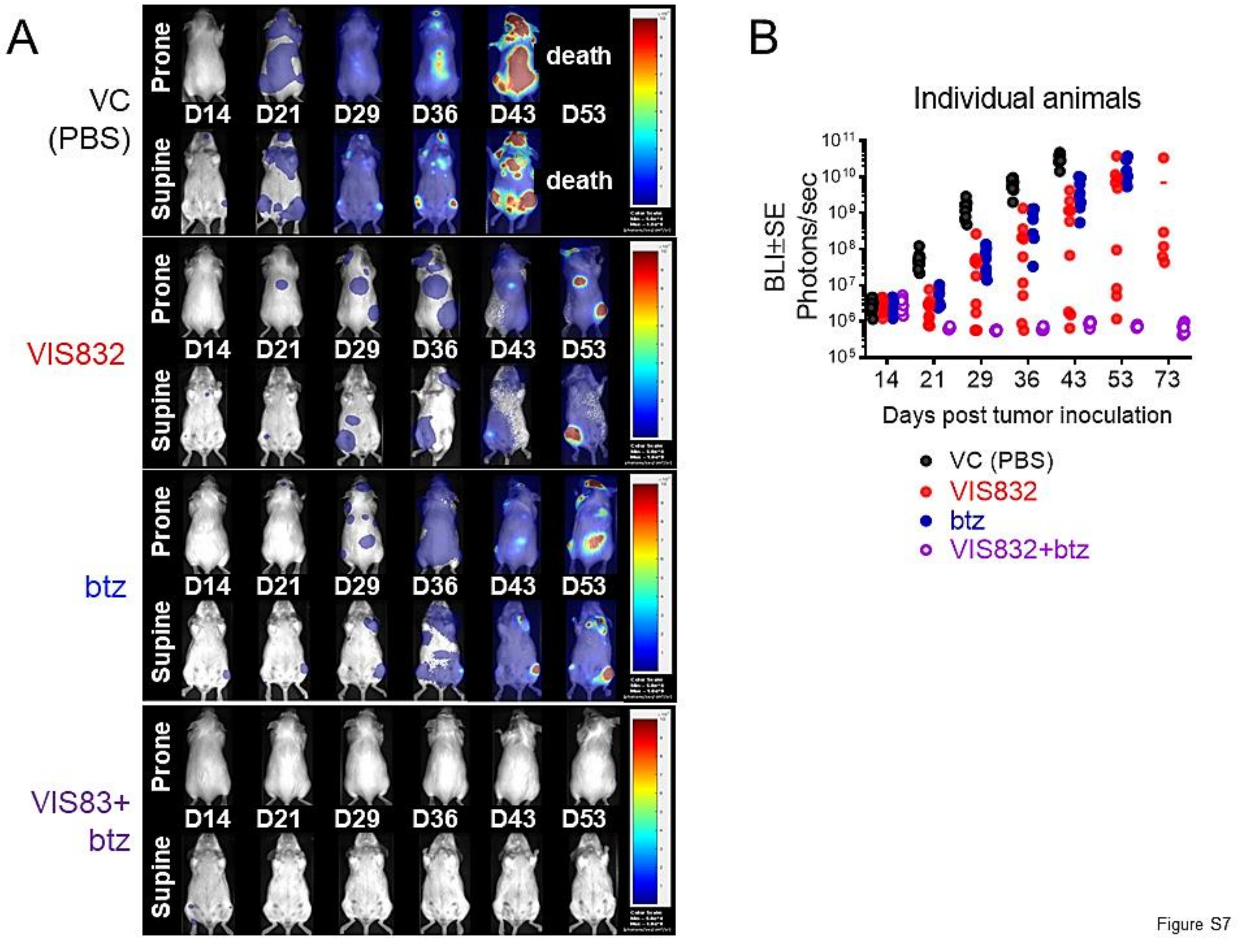

Figure S7. ***In vivo*** **treatment effects followed by changes in BLI in animals**
A, Representative images of individual mice from each group (**Fig. 6**) in the efficacy study, chosen based on their proximity to median BLI at day 36. Image intensity was adjusted for purposes of normalization (min of 1.0e106; max of $3.0x10^8$) and does not imply lack of tumor burden at day 14 (day of staging and prior to first dose). Note that all animals in VC (PBS) group were deceased by day 51. **B**, BLI follow-up in individual mouse (n=9 per group).
